# Thermodynamic Molecular Switch in Sequence-Specific Hydrophobic Interaction: Two Computational Models Compared

**DOI:** 10.1100/tsw.2003.16

**Published:** 2003-03-31

**Authors:** Paul Chun

**Affiliations:** Department of Biochemistry and Molecular Biology, Box 100245, College of Medicine, University of Florida, Gainesville, FL 32610-0245, USA

**Keywords:** sequence-specific hydrophobic interactions, thermodynamic molecular switch, Planck-Benzinger methodology

## Abstract

We have shown in our published work the existence of a thermodynamic switch in biological systems wherein a change of sign in ΔCp°(T)_reaction_ leads to a true negative minimum in the Gibbs free energy change of reaction, and hence, a maximum in the related K_eq_. We have examined 35 pair-wise, sequence-specific hydrophobic interactions over the temperature range of 273–333 K, based on data reported by Nemethy and Scheraga in 1962. A closer look at a single example, the pair-wise hydrophobic interaction of leucine-isoleucine, will demonstrate the significant differences when the data are analyzed using the Nemethy-Scheraga model or treated by the Planck-Benzinger methodology which we have developed. The change in inherent chemical bond energy at 0 K, ΔH°(T_0_) is 7.53 kcal mol compared with 2.4 kcal mol, while ‹t_s_› is 365 K as compared with 355 K, for the Nemethy-Scheraga and Planck-Benzinger model, respectively. At ‹t_m_›, the thermal agitation energy is about five times greater than ΔH°(T_0_) in the Planck-Benzinger model, that is 465 K compared to 497 K in the Nemethy-Scheraga model. The results imply that the negative Gibbs free energy minimum at a well-defined ‹t_s_›, where TΔS° = 0 at about 355 K, has its origin in the sequence-specific hydrophobic interactions, which are highly dependent on details of molecular structure. The Nemethy-Scheraga model shows no evidence of the thermodynamic molecular switch that we have found to be a universal feature of biological interactions. The Planck-Benzinger method is the best known for evaluating the innate temperature-invariant enthalpy, ΔH°(T_0_), and provides for better understanding of the heat of reaction for biological molecules.

## INTRODUCTION

In 1971, T.H. Benzinger[19] proposed a thermal work function to take into account both Boltzmann statistical energy effects and energies of quantum-mechanical bonds. While the latter are usually not altered significantly in macromolecular reactions, it was Benzinger's conjecture that the large-scale and long-range changes of conformation that accompany protein folding or assembly might generate significant energy differences due to the cumulative alteration of their numerous covalent bond structures.

Our 1988[9] and 1994[10] analyses of available and highly precise data on protein folding in several ribonuclease systems, as well as six self-associating protein systems over the temperature range 220–360 K, enabled us to show that the "chemical bond" component of the overall enthalpy was of significant magnitude, and that it was independent of temperature. This analysis demonstrated the validity of the central concept. Our subsequent studies[1,2,3,4,5,6,7,9,10,11,12,13,14,15,16] have shown unequivocally that each macromolecular process has a temperature-independent component of the enthalpy in addition to the traditionally recognized heat capacity of thermodynamics. Specifically, the temperature-independent component of the chemical bond enthalpy is the inherent strength of the bond as measured at 0 K.

In biological interactions, the innate thermodynamic quantities[9,10,11,12,13,14,15,16], in particular the innate temperature-invariant enthalpy, represent differences in chemical bonding energy of products minus reactants, and thus control the heat of reaction at 0 K. The net difference between the temperature-dependent heat capacities of reaction [$\Delta Cp^o(T)$], taken as products minus reactants, dictates how $\Delta H^o_{reaction}$ behaves at high temperatures.

We believe that we have uncovered a type of physical behavior characteristic of living systems that may prove to be remarkably general. It is, of course, known that living systems can live and operate optimally only at a sharply defined temperature, or over a limited temperature range at best. We assert that this implies that basic biochemical macromolecular interactions exhibit a well-defined negative free energy minimum (that is to say, favorable) as a function of temperature. Such a situation is not common in simple chemical systems, where a monotonic change of $\Delta G^o$, $\Delta H^o$, and $K_{eq}$ over an experimental temperature range is typical.

We have found that the critical factor driving the pair-wise hydrophobic interaction of leucine-isoleucine, and 35 similar dipeptides over the temperature range of 273–333 K reported by Nemethy and Scheraga in 1962[8] is a temperature-dependent heat capacity change of reaction, $\Delta Cp^o(T)_{reaction}$, which is positive at low temperature but switches to a negative value at a temperature well below the ambient range. This change of sign of the critically important $\Delta Cp^o(T)_{reaction}$ (product minus reactants) has such significant consequences that we refer to it as a "thermodynamic molecular switch". It determines the behavior patterns of the Gibbs free energy change, and hence a change in the equilibrium constant, $K_{eq}$, and/or spontaneity. Note that $\Delta G^o(T)_{reaction} = -RT \ln K_{eq}$, so that the roles of $\Delta G^o$ and $K_{eq}$ are rigidly coupled. The subsequent, mathematically predictable changes in $\Delta H^o$, $\Delta S^o$, $\Delta W^o$, and $\Delta G^o$ which arise as a result of this thermodynamic molecular switch are demonstrated in this pair-wise, sequence-specific hydrophobic interaction[5,6,7].

Based on Chun's development of the Planck-Benzinger methodology, the change in inherent chemical bond energy at 0 K, $\Delta H^o(T_0)$, is 3.0 kcal $mol^{-1}$ for Leu-Ile, about three times smaller than Nemethy and Scheraga's approach[8]. At $<T_m>$, the thermal agitation energy,

$$\int_0^T \Delta Cp^o(T)dT$$

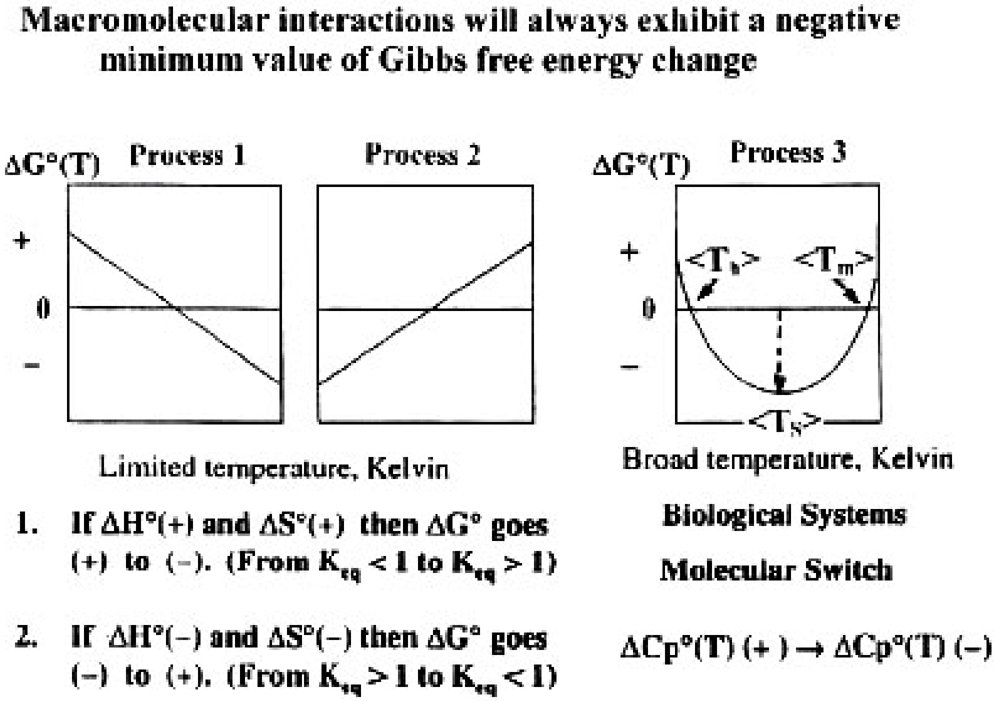

**FIGURE 1**. In all biological interactions, $\Delta H^o(T)$ and $\Delta S^o(T)$ are positive at low temperature. As reaction temperature increases, both $\Delta H^o(T)$ and $\Delta S^o(T)$ become negative, creating negative Gibbs free energy minimum: that is process 1 of the chart goes to process 2, creating process 3 (see Table 1). The change of sign in $\Delta Cp^o\,(T)_{reaction}$ leads to a true negative minimum in the Gibbs free energy of reaction, that is $\Delta Cp^o(+) \rightarrow \Delta Cp^o(-)$, designated as a thermodynamic molecular switch. It determines the behavior patterns of the Gibbs free energy change, and hence a change in the equilibrium constant, $K_{eq}$, and/or spontaneity.

is 12.8 kcal $mol^{-1}$, about five times greater than $\Delta H^o(T_0)$. This pair-wise, sequence-specific hydrophobic interaction is highly similar in its thermodynamic behavior to that of other biological systems, except that the negative Gibbs free energy change minimum at $<T_s>$ occurs at a considerably higher temperature, 355 K compared to about 300 K. The melting temperature, $<T_m>$, is also high, 465 K compared to 343 K in a biological system. The implication is that the negative Gibbs free energy minimum at a well-defined $<T_s>$ has it origin in the hydrophobic interactions, which are highly dependent on details of molecular structure[6,7].

We have shown in our unpublished work the existence of a thermodynamic molecular switch in the interactions of 35 dipeptides over the temperature range of 273–333 K as initially reported by Nemethy and Scheraga[8] and which we have subsequently analyzed by the Planck-Benzinger methodology. In the hydrophobic interaction of each of 35 dipeptide pairs, a change of sign in $\Delta Cp^o(T)_{reaction}$ leads to true negative minimum in the Gibbs free energy of reaction, $\Delta G^o(T)_{reaction}$ and hence a maximum in the related $K_{eq}$, as shown in Fig. 1 and Table 1. Indeed, all interacting biological systems we have examined using the Planck-Benzinger methodology have shown such a thermodynamic molecular switch, suggesting that its existence may be universal[1,2,3,4,5,6,7].

A closer look at a single example, the pair-wise sequence-specific hydrophobic interaction of leucine-isoleucine, will demonstrate the significant differences when the Nemethy-Scheraga data are treated by the Planck-Benzinger methodology.

## THE GIAUQUE FUNCTION AND PLANCK-BENZINGER THERMAL WORK FUNCTION

One form of the free energy function, the Giauque function[20,21,22,23,24],

$$(G_0^o - H_0^o)/T = -\psi^o/T\text{, where } -\psi^o = (G_T^o - H_0^o)$$

has been extensively used in chemistry and physics.

**TABLE 1**
**Enthalpy-Entropy Compensation in Chemical Reactions where ΔG° = ΔH° - TΔS°**

| | ΔG° | ΔH° | ΔS° | TΔS° | Observations |
|---|---|---|---|---|---|
| 1 | – | + | + | – | Entropy-driven process |
| 2 | – | – | – | + | Enthalpy-driven process |
| 3 | + | + | + | – | Entropy-driven at high temperature |
| 4 | – | – | + | – | Reaction always proceeds at all temperatures |

As experimentally observed in interacting biological systems, at low temperature, ΔH° and ΔS° are both positive, becoming negative as temperature increases, whereas ΔG° changes from positive to negative, then reaches a negative value of maximum magnitude at <$T_s$>, and finally becomes positive as temperature increases (Figs. 2 B,D). That is, process 1 goes to process 2, creating cooperative enthalpy-entropy compensation between <$T_h$> and <$T_m$>, where both ΔH°(T)(+) and TΔS°(T)(+) intercept at <$T_h$>. Both ΔH°(T)(–) and TΔS°(T)(–) intercept at <$T_m$>. This process is illustrated schematically in Fig. 1.

An equivalent formulation has recently found application in biochemical literature as the Planck-Benzinger thermal work function[1,2,3,4,5,6,7,9,10,11,12,13,14,15,16], where the application to a given situation is quite different. Here the Planck-Benzinger thermal work function, $\Delta W(T) = \Delta H(T_0)–\Delta G(T)$[19], represents the strictly thermal components of any intra- or intermolecular bonding term, that is, energy other than the inherent difference of the 0 K portion of the interaction energy. The latter is the only energy term in constant pressure processes at absolute 0 K. Thus ΔW(T) expresses completely the thermal energy difference of the process involved. Application of the Planck-Benzinger thermal work function permits the separation of 0 K energy differences and energy differences associated with heat capacity integrals for a fuller understanding of reaction energies.

## MEASURING ENTHALPY VALUES

Enthalpies of reaction are frequently measured at or near room temperature (298 K) for a variety of theoretical and practical reasons, for instance, the relationship between $\Delta H^o_{reaction}$ and the temperature coefficient of the equilibrium constant, $K_{eq}$, $d\ln K_{eq}/d(1/T) = -\Delta H^o(T)/R$. Kirchhoff[18,24,25,26,27] stated

$$\Delta H^o_{298} = \Delta H^o(T_0) + \int_0^T \Delta Cp^o dT$$

where this last term represents the thermal agitation energy (heat capacity integrals), while the constant term $\Delta H^o(T_0)$ represents the enthalpy of reaction at 0 K. For small molecules, reaction enthalpies are often obtained around room temperature, and the heat of reaction is estimated in terms of the innate temperature-invariant enthalpy, $\Delta H^o(T_0)$.

T.L. Cottrell[27] pointed out 40 years ago that $\Delta H^o_{298}$ and $\Delta H^o(T_0)$ differ only by about 1% in small molecules, but in 1971 T.H. Benzinger made the crucial observation that this difference is large in biological macromolecules due to the large magnitude of the heat capacity integrals (thermal agitation energy). In other words, for small molecules, $[(\Delta H^o_{298} - \Delta H^o(T_0)]$ is a correction of only a few percent, whereas for biological macromolecules, the heat capacity integrals can be large, from 10% up to 50% of the total heat of reaction. At present the scientific literature provides no “silver bullet”, that is no highly accurate method for evaluating $[(\Delta H^o_{298} - \Delta H^o(T_0)]$ in large biological macromolecules; however, Chun's work[1,2,3,4,5,6,7,9,10,11,12,13,14,15,16] has extensively addressed the problem.

## THE PLANCK-BENZINGER APPROACH

In order to analyze the thermodynamic processes operating in a pair-wise hydrophobic interaction such as leucine-isoleucine, it is necessary to extrapolate the thermodynamic parameters over a much broader temperature range. The enthalpy, entropy, and heat capacity terms are evaluated as partial derivatives of the Gibbs free energy function defined by Helmholtz-Kelvin's expression[25,26].

$$\partial \Delta G(T)/\partial T = -\Delta S(T), \qquad \{\partial \Delta G(T)/T\}/\partial T = -\Delta H(T)/T^2$$

$$\partial \Delta H(T)/\partial T = \Delta Cp(T), \qquad \partial \Delta S(T)/\partial T = \Delta Cp(T)/T$$

In continuing studies on dozens of interacting protein systems, it has been shown in our laboratory that the third-order polynomial function provides a good fit in the temperature range accessible in biochemical systems. In fact, it is shown to be correct in the low-temperature limit. The rationale for selecting the linear and nonlinear third-order($T^3$ model) polynomial functions for $\Delta G^o(T) = \alpha + \beta T^2 + \gamma T^3$ (macromolecular interaction) and $\Delta H(T) = \alpha + \beta T^3 + e^{\gamma T}$ (protein unfolding) are found in the fundamentals of relevant quantum theory[25].

The constraints for this fitted model of the Gibbs free energy change as a function of temperature are these: the enthalpy and Gibbs free energy must intersect at 0 K at a positive value and the slope must be zero where $\Delta G^o(T_0) = \Delta H^o(T_0)$ at 0 K as $T\Delta S^o$ approaches zero. The cubic term is necessitated by quantum mechanical considerations, and the presence of the quadratic term is dictated by the data rather than theory[1,2,3,4,5,8,9,10,11,12,13,14,15].

## DETERMINATION OF THE GIBBS FREE ENERGY CHANGE AS A FUNCTION OF TEMPERATURE

Nemethy and Scheraga’s theoretical treatment of hydrophobic interaction[8,28] based on a statistical mechanical theory of the thermodynamic properties of liquid water[29] and of aqueous solutions of hydrocarbon[30] led to determination of the maximum strength between isolated hydrophobic side chains. $\Delta G^o(T)$ data for a pair-wise hydrophobic interaction of maximum strength were computed from Nemethy and Scheraga's Table II[8] based on $T^2$ model of $\Delta G^o = a + bT + cT^2$ over a temperature range of 273–333 K, knowing the polynomial coefficients a, b, c[8,28]. Other thermodynamic parameters were are follows:

$$\Delta G^o = a + bT + cT^2$$
$$\Delta H^o(T) = a - cT^2$$
$$T\Delta S^o(T) = -bT - 2cT^2$$
$$\Delta Cp^o = -2cT$$

Values for the Gibbs free energy change as a function of temperature were computed for the sequence-specific hydrophobic interactions of leucine-isoleucine and compared with values determined using our $T^3$ model of $\Delta G^o(T) = \alpha + \beta T^2 + \gamma T^3$[5].

## COMPUTING HYDROPHOBIC INTERACTION BY THE PLANCK-BENZINGER METHOD

In order to extrapolate down to 0 K, it is necessary to consider the normal solution states of molecules. Here the 0 K limit would presumably refer to the glassy condition[1,2,3,4,5,6,7,13,14,15], that is a condition with all thermal agitation frozen out, but retaining the general physical chemical properties of solution – since a pure crystalline form of macromolecules is rarely encountered in practice.

The approach that we follow requires exact determination of $K_{eq}$ for the relevant biochemical processes as a function of absolute temperature. Of critical importance, however, is the use of precise, correctly formulated expressions for $\Delta Cp^o_{reaction}$ as a function of temperature as shown in Eq. 3. In this treatment, the Gibbs free energy data, as shown in Fig. 2A, were fitted to a three-term linear polynomial function in the 273–333 K temperature range, the range in which computations have been conducted as shown in Table 2.

$$\Delta G^o(T) = \alpha + \beta T^2 + \gamma T^3 \qquad (1)$$

Once evaluated as shown in Fig. 2A (see Table 2), the coefficients α, β, and γ were fitted to other thermodynamic parameters. $\Delta H^o(T)$, $\Delta Cp^o(T)$, $T\Delta S^o(T)$, and $\Delta W^o(T)$ are defined by Helmholtz-Kelvin expression as follows:

$$\Delta H^o(T) = \alpha - \beta T^2 - 2\gamma T^3 \qquad (2)$$

$$\Delta Cp^o(T) = -2\beta T - 6\gamma T^2 \qquad (3)$$

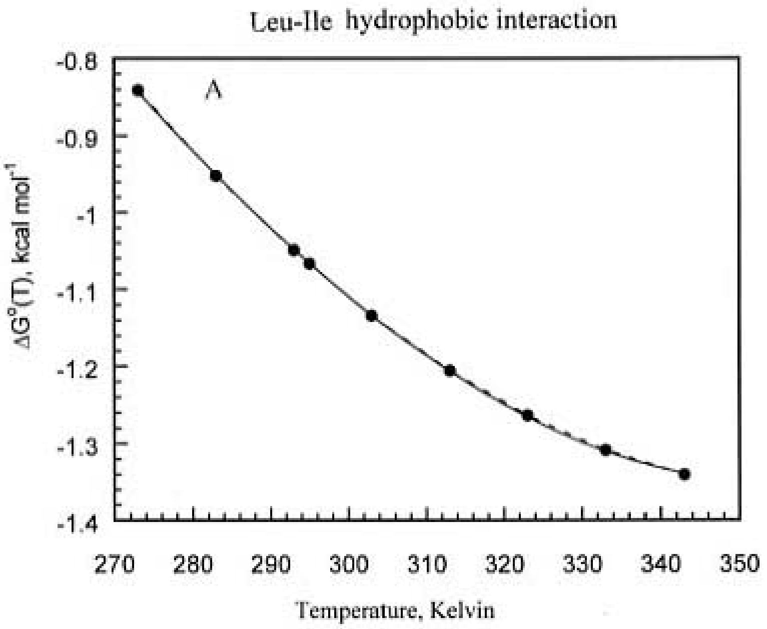

**FIGURE 2A**. Thermodynamic plot of the standard Gibbs free energy change of Leu-Ile hydrophobic interaction. Values for $\Delta G^o(T)$ as a function of temperature were computed from Nemethy and Scheraga's Table II[8] in the temperature range 273–333 K, using the general linear model ($T^3$ model) procedure of statistical analysis of IMSL subroutine. The solid line represents fitted data. F = 0.0001, thus the goodness of fit of the experimental data was 99.9% or better in each case[31,32].

**TABLE 2**
**Values of $\Delta H^o(T_0)$ and Expansion Coefficients for the Hydrophobic Interaction of Leu-Ile, Comparing the Planck-Benzinger and Nemethy-Scheraga Approaches**

| Hydrophobic Interactions | $\alpha$,[$\Delta H^o(T_0)$] [kcal mol$^{-1}$] | $\beta$ (kcal mol$^{-1}$ K$^{-2}$) | $\gamma$ (kcal mol$^{-1}$ K$^{-3}$) |
|---|---|---|---|
| Leu-Ile (Planck-Benzinger) | 2.4183 | $-8.9841 \times 10^{-5}$ | $1.6883 \times 10^{-7}$ |
| Leu-Ile (Nemethy and Scheraga) | 7.2560 | $-4.7830 \times 10^{-2}$ | $6.5654 \times 10^{-5}$ |

*Note*: Compiled using the general linear model procedure analysis of IMSL subroutine adapted for use in KaleidaGraph 3.5.1. Values for these $\Delta H^o(T_0)$ vary by less than 0.01%. F = 0.0001, thus the goodness of fit of the computed data was 99.99% or better in each case. Each data point between 0 and 350 K was evaluated with extrapolation of F- statistics[31,32].

Leu-Ile: Chisq = $1.6329 \times 10^{-5}$; $R^2$ = 0.99996; SD = NA; PR >F = 0.0001. Leu-Ile (Nemethy and Scheraga approach)[8,28]: Chisq = $4.6406 \times 10^{-7}$; $R^2$ = 1.0000; SD = NA; PR >F = 1.0000.

$$T\Delta S^o(T) = -2\beta T^2 - 3\gamma T^3 \quad (4)$$

$$\Delta W^o(T) = -\beta T^2 - \gamma T^3 \quad (5)$$

To extrapolate down to 0 K, it is necessary to consider normal solution states of macromolecules. Here the 0 K limit would presumably refer to the glassy condition[12], as described earlier.

Data for the Gibbs free energies were fitted to a model and the derived quantities for other thermodynamic parameters were calculated and plotted using the International Mathematical Subroutine Library (IMSL) software for linear and nonlinear polynomial regression analysis. Each equation was interactively executed in steps of 1 K, and the values plotted and overlaid for each set of experimental conditions. This IMSL subroutine was adapted for use in KaleidaGraph 3.5.1 and each data point was evaluated with extrapolation of F-statistics in an IBM personal computer[31,32], as shown in Figs. 2B-D.

A built-in restriction in the extrapolation procedure is that the values for ΔHº(T) and ΔGº(T) determined from the polynomial functions intersect at 0 K with zero slope on a thermodynamic plot, thus obeying Planck's definition of the Nernst heat theorem[17,25]. By our definition, the value of $\Delta H^o(T_0)$ will be positive. Other polynomial functions failed to meet all three restrictions of ΔHº(T) and ΔGº(T) intersecting at 0 K with zero slope, and $\Delta H^o(T_0)$ being positive and thus were discarded.

It is clear that a temperature-dependent model simpler than the third-order Gibbs polynomial model ($T^3$ model) cannot be used at low temperature, and has been found to be unacceptable at room temperature; therefore it would be reasonable to apply a more complex model in the intervening temperature region only if the facts demand a more complex fit. In fact, the facts do not require a different function; the model as described has been found to have both strong correlative power and very good predictive power.

The fitted thermal data [ΔGº(T), ΔHº(T), ΔWº(T), TΔSº(T), and ΔCpº(T)] are reasonable not only over the measured experimental range (near room temperature) but also in the low temperature limit. The fitting curves do nothing strange in the experimentally inaccessible region, but rather smoothly approach the low temperature limit. The thermal dependency presented here is the best (and essentially the only) approach known.

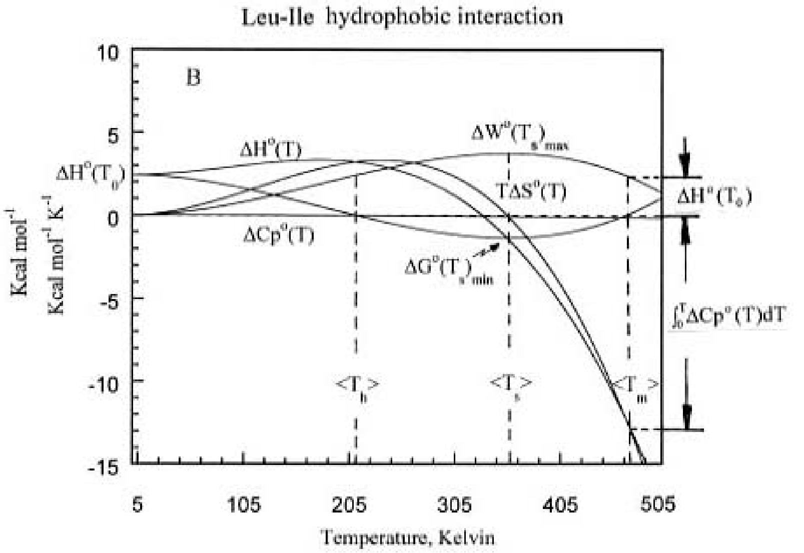

**FIGURE 2B.** Thermodynamic plot of the Planck-Benzinger thermal work function for Leu-Ile hydrophobic interaction. Each data point between 0 and 500 K was evaluated with extrapolation of F-statistics. The solid line represents fitted data, F = 0.0001, thus the goodness of fit of the experimental data was 99.9% or better in each case.

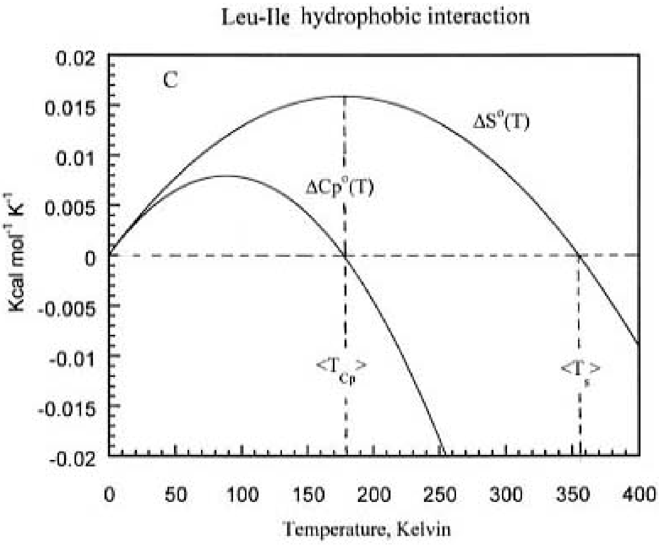

**FIGURE 2C.** A close-up view of a portion of Leu-Ile hydrophobic interaction as shown in Fig. 2B, over temperature range of 0–400 K, with the magnitude of the Y-axis reduced to 0.02 to –0.02 kcal $mol^{-1}$. The thermodynamic molecular switch occurs when $\Delta Cp^o(T) = 0$ at $<T_{Cp}> = 180$ K, $\Delta Cp^o(T)$ changes sign from positive to negative, while $\Delta S^o(T) = 0$ or $T\Delta S^o(T)$ changes from positive to negative at $<T_s> = 355$ K.

In the thermodynamic methods based on our development of Planck-Benzinger methodology[1,2,3,4,5,6,7,8,9,10,11,12,13,14,15], computed values must be in agreement with experimentally obtained $K_{eq}$ values [and therefore with values of $\Delta G^o(T)$ of reaction] of biological systems over a temperature range of 273–343 K.

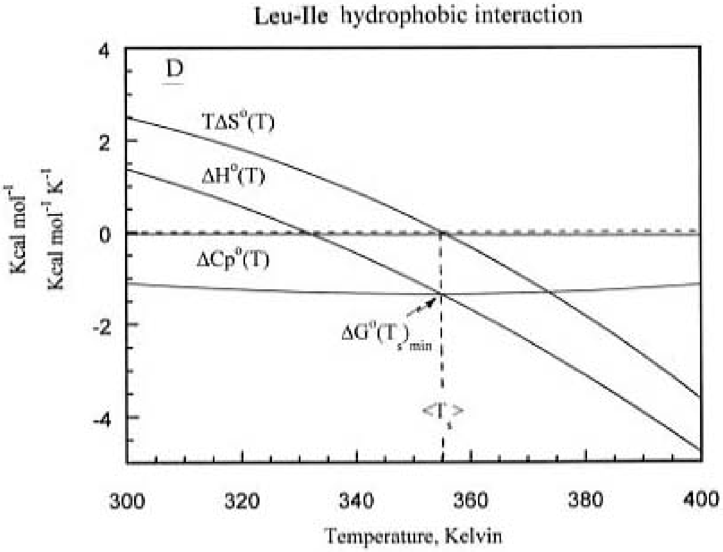

**FIGURE 2D**. A close-up view of a portion of Leu-Ile hydrophobic interaction as shown in Fig. 2B, over the temperature range of 300–400 K, with the magnitude of the y-axis reduced to 4 to –4 kcal $mol^{-1}$ $K^{-1}$.

## WHY IS HELMHOLTZ-KELVIN'S EXPRESSION CONSIDERED TO BE A CONTINUOUS FUNCTION?

It is true that a change of phase for a specific chemical substance causes an infinite discontinuity in Cp, a finite discontinuity in S and H, and an abrupt change in the slope of G vs. T. Similar relevant data for phase changes in biological reactions are generally lacking; in our work the reference condition at 0 K is taken as a glassy solid. In this case, all thermodynamic functions are continuous down to 0 K.

Our evaluation of the innate temperature-invariant enthalpy for hydrogen-bonded water, in equilibrium with nonhydrogen bonded water molecules, is based on Helmholtz free energy data reported by Nemethy and Scheraga[29]. The entropy of the system appears to remain independent of temperature, suggesting that there is no significant temperature-dependent difference in the degree of orientation between unbound and hydrogen-bound water molecules in equilibrium in the system. A similar conclusion could be reached based on the dielectric relaxation of water as a function of temperature, as reported by Collie et al.[33]. This implies that there is a nonzero entropy difference for the transformation between water monomer and n-mer at 0 K (unpublished results[13]). As with most small molecules, the thermal agitation energy is minimal. $\Delta W(T) = T\Delta S(T)$, $\Delta H(T) = \Delta H(T_0)$ over the entire temperature range from 0 K to the temperature of interest. As already noted, the assumed reference condition is glassy ice at 0 K, which probably does have a small $\Delta S(T_0)$. However, $T\Delta S^o(T_0) = 0$, in any event. In the case of $\Delta G^o(T)$ of formation, phase transition is indeed important. When dealing with $\Delta G^o(T)$ of reaction, no phase transition is taking place. In this case, all thermodynamic functions are continuous.

## ANALYSIS OF PLANCK-BENZINGER THERMAL WORK FUNCTION

A plot of the Gibbs polynomial function, $\Delta G^o(T) = \alpha + \beta T^2 + \gamma T^3$, as a function of temperature exhibits an initial value of zero for $\Delta G^o(T)$ at $<T_h>$, a negative minimum value for $\Delta G^o(T)$ at $<T_s>$, and the $\Delta G^o(T)$ value again reaches zero at $<T_m>$. Here $<T_s>$ is the stable temperature at which $T\Delta S^o(T) = 0$, $<T_m>$ is the melting temperature; and $<T_h>$ is the harmonious temperature at which $\Delta G^o(T)$ is zero, $\Delta Cp^o(T)$ approaches zero and $T\Delta S^o(T)$ reaches a positive maximum (see Figs. 2B-C). Values of the innate temperature-invariant enthalpy at $<T_h>$, $<T_s>$, $<T_m>$ are compared with those at 0 K as shown in Figs. 2B-D (see Table 3). The change in inherent chemical bond energy at 0 K, $\Delta H^o(T_0)$, is 2.4 for Leu-Ile. In this pair, as with others we have examined, the value of $\Delta H^o(T_0)$ decreases as the length of the hydrophobic side chain decreases. $<T_s>$ remains constant at 355 K and $<T_m>$ is about 470 K in each system. It is apparent that the strength and stability of the hydrophobic interaction is determined by the packing density of the side chains. At $<T_m>$, the thermal agitation energy is about five times greater than $\Delta H^o(T_0)$. Additionally, the thermal agitation energy for the same series of pair-wise sequence-specific hydrophobic interaction, evaluated at $<T_m>$, decreases in the same order, that is as the length of the side chain decreases.

1. $\Delta H^o(T_0) = \Delta W^o(T_h)$, $\Delta G^o(T) = 0$ at $<T_h>$. Both $\Delta H^o(T)(+)$ and $T\Delta S^o(T)(+)$ intercept at $<T_h>$.

2. $\Delta H^o(T_0) = \Delta W^o(T_s)_{max} + \Delta G^o(T_s)_{min}$, $\Delta H^o(T_s) = \Delta G^o(T_s)_{min}$ at $<T_s>$

3. $\Delta H^o(T_0) = \Delta W^o(T_m)$, $\Delta G°(T) = 0$ at <$T_m$> and one can define the heat of reaction as $\Delta H^o(T) = \Delta H^o(T_0) + \int_{T_0}^{T} \Delta Cp^o(T)dT$. Both $\Delta H^o(T)$(–) and $T\Delta S^o(T)$( –) intercept at <$T_m$>.

4. $\Delta H^o(T_0)$ is evaluated at 0 K.

The values of $\Delta W^o(T)$ and $\Delta G^o(T)$ exhibit a positive maximum and negative minimum, respectively, at <$T_s$>; therefore, the innate temperature-invariant enthalpy, $\Delta H^o(T_0) = \Delta W^o(T_s)_{max} + \Delta G°(T_s)_{min}$ at <$T_s$>. The innate temperature-invariant enthalpy at the melting temperature is, by the integrated Kirchhoff expression, $\Delta H^o(T) = \Delta H^o(T_0) + \int_0^T \Delta Cp^o(T)dT$, where $\Delta H^o(T)$ and $T\Delta S^o(T)$ are of the same magnitude, $\Delta W^o(T) = \Delta H^o(T_0)$ and $\Delta G^o(T)$ approaches zero. The nature of the biochemical thermodynamic compensation that takes place between <$T_h$> and <$T_m$> may be characterized by evaluating $\Delta H^o(T_0)$ and the heat capacity integrals (see Figs. 2B-D and Table 3).

**TABLE 3**
**Comparison of $\Delta H^o(T_0)$ at <$T_h$>, <$T_s$>, <$T_m$> and 0 K for a Pair-Wise Hydrophobic Interaction**

| Substitution | $\Delta H°(T_0)$ at <$T_h$> (kcal mol$^{-1}$) | $\Delta H°(T_0)$ at <$T_s$> (kcal mol$^{-1}$) | $\Delta H°(T_0)$ at <$T_m$> (kcal mol$^{-1}$) | $\Delta H°(T_0)$ at 0 K (kcal mol$^{-1}$) | <$T_h$> | <$T_s$> (K) | <$T_m$> | $\int_{T_0}^{T} \Delta Cp^o dT$ |
|---|---|---|---|---|---|---|---|---|
| Leu-Ile (Planck-Benzinger) | 2.39 | 2.41 | 2.39 | 2.41 | 210 | 355 | 465 | -12.81 ± 0.02 |
| Leu-Ile (Nemethy-Scheraga)[8] | 7.53 | — | — | — | 233 | 365 | 497 | -8.49 ± 0.80 |

*Note*: Compiled using the general linear model procedure of statistical analysis of IMSL subroutine adapted for use in KaleidaGraph 3.5.1. Values for these four $\Delta H°(T_0)$ vary by less than 0.2%. F = 0.0001, thus the goodness of fit of the experimental data was 99.9% or better in each use. Observed values for <$T_m$> are theoretical values.

As shown in Table 1, in this hydrophobic interaction, at low temperature, $\Delta H^o(T)$ and $\Delta S^o(T)$ are both positive (entropy-driven process), becoming negative as temperature increases (enthalpy-driven process), whereas $\Delta G^o(T)$ changes from positive to negative then reaches a negative value of maximum magnitude at <$T_s$>, and finally becomes positive as temperature increases (see Figs. 2B-D). That is, process 1 goes to process 2, creating cooperative enthalpy-entropy compensation between <$T_h$> and <$T_m$>, as shown in Table 1 and Fig. 1.

## NEMETHY AND SCHERAGA'S METHOD OF ANALYSIS

Fitting the $T^2$ model of $\Delta G^o = a + bT + cT^2$, for the Gibbs free energy as a function of temperature of Nemethy and Scheraga[8,28] and our $T^3$ model of $\Delta G^o(T) = \alpha + \beta T^2 + \gamma T^3$, based the Planck-Benzinger methodology, it is apparent from Fig. 3A that both models fit the theoretically computed $\Delta G^o(T)$ data equally well over the temperature range of 273–333 K. As shown in Fig. 3B, the values for $\Delta H^o(T)$ and $\Delta G^o(T)$ determined from a linear $T^2$ polynomial function do not intersect at 0 K with zero slope and thus are inconsistent with Planck's definition of the Nernst heat theorem[17]. The value of $\Delta H^o(T_0)$, shown in Table 3, was found to be 7.53 kcal mol$^{-1}$, five times greater then $\Delta H^o(T_0)$ obtained by the Planck-Benzinger approach. The thermal agitation energy was –8.45 kcal $mol^{-1}$, slightly lower than that obtained by our approach (see Fig. 3B).

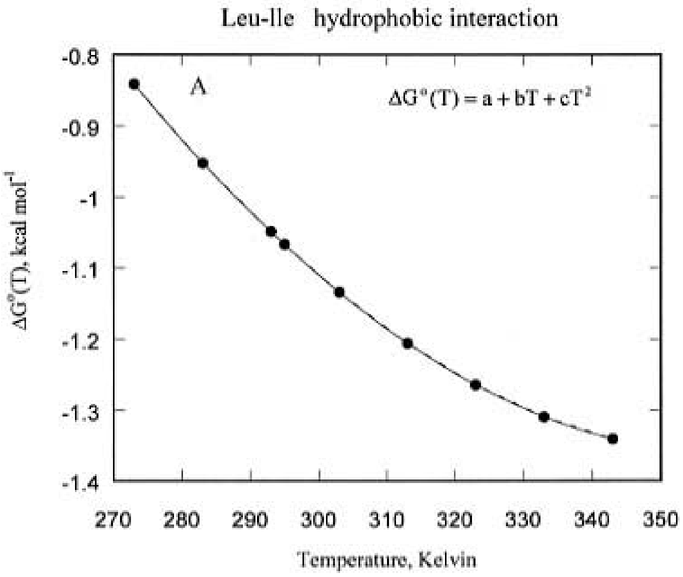

**FIGURE 3A**. Thermodynamic plot of the standard Gibbs free energy change of Leu-Ile hydrophobic interaction. Values for $\Delta G^o(T)$ as a function of temperature were computed from Nemethy and Scheraga's Table II[8] in the temperature range 273–333 K, using the general linear polynomial model ($T^2$ model: $\Delta G^o(T) = a + bT + cT^2$) procedure of statistical analysis of IMSL subroutine. The solid line represents fitted data. F = 0.0001, thus the goodness of fit of the experimental data was 99.99% or better in each case.

In Nemethy and Scheraga's analysis of thermodynamic parameters, fit of the data for heat capacity vs. temperature when extrapolated to 0 K is not fully adequate, as shown in Figs. 3C-D, therefore an unacceptable prediction of entropy is to be expected. Furthermore, no thermodynamic molecular switch is observed, rather $\Delta Cp^o(–) \rightarrow \Delta Cp^o(–)$ (see Table 4).

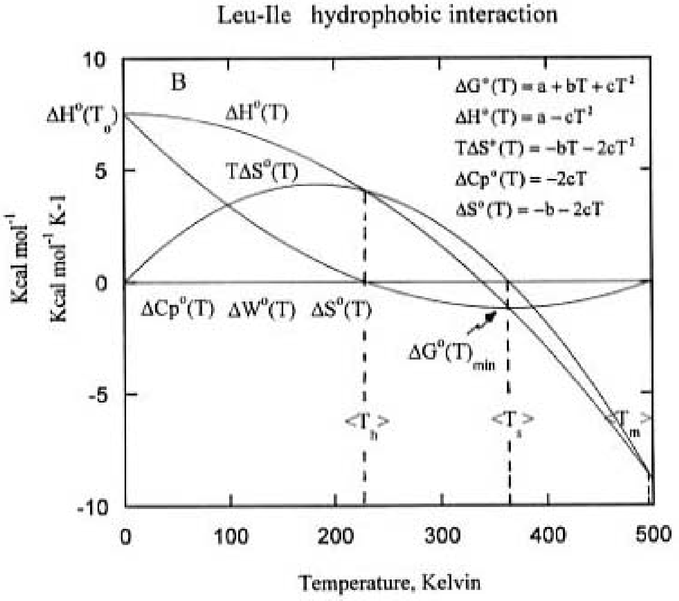

**FIGURE 3B.** Thermodynamic plot of Nemethy-Scheraga $T^2$ model for Leu-Ile hydrophobic interaction. Each data point between 0 and 500 K was evaluated with extrapolation of F-statistics[31,32]. The solid line represents fitted data, F = 0.0001, thus the goodness of fit of the experimental data was 99.99% or better in each case. The values for ΔH°(T) and ΔG°(T) determined from a linear $T^2$ polynomial function do not intercept at 0 K with zero slope and thus are inconsistent with Planck's definition of the Nernst heat theorem[16].

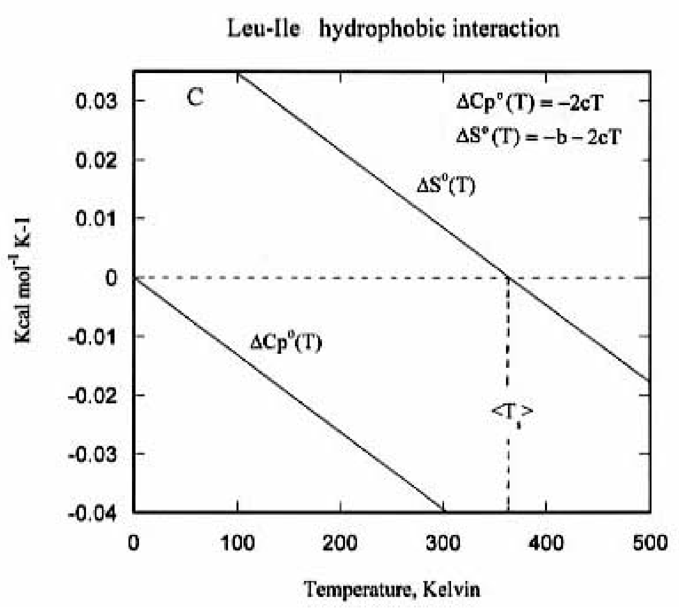

**FIGURE 3C.** A close-up view of a portion of Leu-Ile hydrophobic interaction as shown in Fig. 3B, over temperature range of 0–500 K, with the magnitude of the y-axis reduced to 0.03 to –0.04 kcal $mol^{-1}$. No thermodynamic molecular switch is observed.

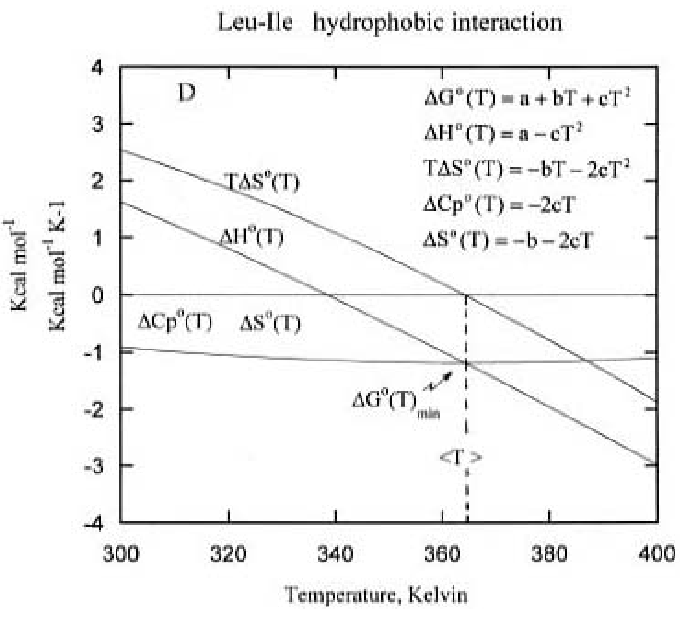

**FIGURE 3D.** A close-up view of a portion of Leu-Ile hydrophobic interaction as shown in Fig. 3B, over the temperature range of 300–400 K, with the magnitude of the y-axis reduced to 4 to –4 kcal $mol^{-1}$ $K^{-1}$.

**TABLE 4**
**Thermodynamic Molecular Switch in $\Delta Cp^o(T)$ and $\Delta S^o(T)$**

| Hydrophobic Interaction | Thermodynamic Quantities | Molecular Switch at Temperature, K | Effect on Sign of $\Delta Cp^o(T)$ and $\Delta S^o(T)$ |
|---|---|---|---|
| (Planck-Benzinger) Leu-Ile | $\Delta Cp^o(T) = 0$ at $<T_{Cp}>$ | 180 | (+) → (–) |
| | $\Delta S^o(T) = 0$ at $<T_s>$ | 355 | (+) → (–) |
| (Nemethy-Scheraga) Leu-Ile | No thermodynamic switch | | $\Delta Cp^o(T) = 0$ at 0 K (–) → (–) |
| | | | $\Delta S^o(T) = +15.7$eu at 0 K (+)→ (–) at 365 K |

## THE BEHAVIOR OF THE GIBBS FREE ENERGY FUNCTION IN INTERACTING BIOLOGICAL SYSTEMS

$\Delta H^o(T)$ and $\Delta S^o(T)$ are simple fundamental thermodynamic functions. In each case the respective value at a given temperature is determined in a straightforward way using the following expression:

$$[\Delta H^o_T - \Delta H^o(T_0)] = \int_0^T \Delta Cp^o(T), \text{ whereas } \Delta S^o_T = \int_0^T (\Delta Cp^o / T) dT$$

Note that the value of the enthalpy change at 0 K, $\Delta H^o(T_0)$, is important, but distinct and separate from the thermal agitation term. Many authors have entirely ignored this, particularly when dealing with biological systems.

**TABLE 5**
**Thermodynamic Molecular Switch in Gibbs Free Energy Change**

| Self-Association Reaction | Thermodynamic Quantities | Molecular Switch at Temperature, K | Effect on Sign | |
|---|---|---|---|---|
| | | | $\Delta G^o(T)$ | $\Delta H^o(T)$ |
| (Planck-Benzinger) | $\Delta G^o(T)$ at $<T_h>$ | 215 | (+)→ (–) | |
| Leu-Ile | $\Delta G^o(T)$ at $<T_s>$ | 355 | Negative minimum | |
| | $\Delta G^o(T)$ at $<T_m>$ | 465 | (–) → (+) | |
| | $\Delta H^o(T)$ | 330 | | (+) → (–) |
| (Nemethy-Scheraga) | $\Delta G^o(T)$ at $<T_h>$ | 233 | (+) → (–) | |
| Leu-Ile | $\Delta G^o(T)$ at $<T_s>$ | 365 | Negative minimum | |
| | $\Delta G^o(T)$ at $<T_m>$ | 497 | (–) → (+) | |
| | $\Delta H^o(T)$ | 343 | | (+) → (–) |

In contrast, the Gibbs free energy function is a composite quantity, defined as a trade-off of $\Delta H^o(T)$ and $\Delta S^o(T)$ terms:

$$\Delta G^o(T) = \Delta H^o(T) - T\Delta S^o(T)$$
$$\Delta G^o(T) = \Delta H^o(T_0) + \int_0^T \Delta Cp^o dT - T\int_0^T (\Delta Cp^o / T)dT$$

With proper rearrangement, this equation also yields the Giauque and Planck-Benzinger thermal work functions[1,2,3,4,5,6,7,9,10,11,12,13,14,15,16]. In consequence $\Delta G^o(T)$ displays an interesting variety of behavior patterns as the temperature changes. $\Delta G^o$ can change sign ($K_{eq}$ from <1 to >1 or vice versa ) only if $\Delta H^o$ and $\Delta S°$ remain of the same sign (see Tables 1, 5, and Fig. 1). That is to say:

1. If $\Delta H^o$ is (+) and $\Delta S^o$ is (+) then $\Delta G^o$ goes from (+), unfavorable to (–), which is favorable. (From $K_{eq}$ <1 to $K_{eq}$ >1.)
2. If $\Delta H^o$ is (–) and $\Delta S^o$ is (–) then $\Delta G^o$ goes from (–), favorable to (+), which is unfavorable. (From $K_{eq}$ >1 to $K_{eq}$ <1.)

In all biological interactions, $\Delta H^o(T)$ and $\Delta S^o(T)$ are positive at low temperature. As reaction temperature increases, both $\Delta H^o(T)$ and $\Delta S^o(T)$ become negative; that is scheme (1) of the chart goes to scheme (2): $\Delta H^o(+)$ and $\Delta S^o(+) \rightarrow \Delta H^o(-)$ and $\Delta S^o(-)$, creating a negative Gibbs free energy minimum as shown in Fig. 1 (see Table 1). In this thermodynamic switch unique to and characteristic of hydrophobic interaction, $\Delta Cp^o(T)$ changes from positive to negative at $<T_{Cp}>$, shown in Fig. 2C. As seen in Table 4, the Gibbs free energy change switches sign at $<T_h>$ and $<T_m>$, where $\Delta G^o(T) = 0$, and reaches a negative minimum at $<T_s>$. The $<T_s>$ value is 355 K at $T\Delta S^o(T) = 0$ (see Table 1 and Figs. 2B-D).

$$\Delta G^o(T)(+) \rightarrow \Delta G^o(T_s)_{min}(-) \rightarrow \Delta G^o(T)(+)$$

$$\Delta S^o(+) \rightarrow \Delta S^o(-) \text{ where } \Delta S^o = 0 \text{ at } <T_s>$$

At temperature $<T_s>$, a simple algebraic sum of $\Delta W^o(T_s)$ and $\Delta H^o(T_s)$ yields the value of $\Delta H^o(T_0)$, i.e., $\Delta H^o(T_0) = \Delta W^o(T_s)_{max} + \Delta H^o(T_s)$ (see Fig. 2B). At $<T_s>$ there exists an optimal balance of $\Delta H^o(T_s) = \Delta G^o(T_s)_{min}$ and $T\Delta S^o(T)$, so there will be minimum negative Gibbs free energy change and the maximum work can be accomplished.

## THERMODYNAMIC MOLECULAR SWITCH IN SEQUENCE-SPECIFIC HYDROPHOBIC INTERACTIONS

For the pair-wise, sequence-specific hydrophobic interaction of Leu-Ile we have examined, $\Delta Cp^o(T)$ reaches a maximum at 90 K, while the sign changes from positive to negative at $<T_{Cp}>$ = 180 K, the temperature at which $\Delta Cp^o(T) = 0$. Similarly $\Delta S^o(T)$ reaches a maximum at 180 K, while the sign changes from positive to negative at $<T_s>$ = 355 K, the temperature at which $T\Delta S^o(T) = 0$

and the Gibbs free energy change reaches a negative mimimum as shown in Figs. 2C-D (see Table 5).

The significance of the negative Gibbs free energy minimum in equilibria of sequence-specific dipeptides and on a wider scope, of biological systems, is that one is dealing with a true condition of stability, which is the maximum $K_{eq}$ of the reaction. It is no surprise that the temperature of maximum stability should be found over a broad temperature range in biological systems, since such systems are known to exist optimally from arctic temperatures to several hundred degrees Centigrade in fumerals of the ocean floor.

These data demonstrate that the critical factor is a temperature-dependent heat of reaction, $\Delta Cp^{o}(T)$, which is positive at low temperature but switches to a negative value at $<T_{Cp}> = 180$ K, the ambient temperature range. Note that this thermodynamic switch in the sign of $\Delta Cp^{o}(T)_{reaction}$ determines the behavior patterns of the Gibbs free energy change and hence a change in the equilibrium constant, $K_{eq}$, and/or spontaneity, as shown in Figs. 2C-D. The subsequent, mathematically predictable changes in $\Delta H^{o}(T)$, $\Delta S^{o}(T)$, $\Delta W^{o}(T)$, and $\Delta G^{o}(T)$ give rise to the observed behavior patterns in pair-wise, sequence-specific hydrophobic interaction of dipeptides.

It is clear that nonlinear temperature dependence of $\Delta Cp^{o}(T)_{reaction}$ is the essence of the thermodynamic molecular switch. In the present instance, the hydrophobic interactions seem to be the best candidates for such behavior. At present the ultimate range of applicability of the thermodynamic molecular switch concept is not known.

In the pair-wise sequence-specific hydrophobic interaction of Leu-Ile, the value of $\Delta H^{o}(T_0)$ is 2.40 kcal $mol^{-1}$. It is apparent from results using the Planck-Benzinger approach that the strength and stability of the hydrophobic interaction is determined by the packing density of the side chains. At $<T_m>$, the thermal agitation is about five times greater than $\Delta H^{o}(T_0)$ in these systems. Additionally, the thermal agitation energy for the same series, evaluated at $<T_m>$, decreases in the same order, which is as the length of the side chain decreases.

This pair-wise, sequence-specific dipeptide hydrophobic interaction is highly similar in its thermodynamic behavior to that of other biological systems, except that the negative Gibbs free energy change minimum at $<T_s>$ occurs at a considerably higher temperature, 355 K compared to about 300 K. The melting temperature, $<T_m>$, is also high, 470 K compared to 343 K in a biological system. The implication is that the negative Gibbs free energy minimum at a well-defined $<T_s>$ has its origin in the sequence-specific hydrophobic interactions, which are highly dependent on details of molecular structure. Since detailed statistical mechanical computations on our system have not been attempted, no further clarification can be provided at this time.

In addition to the specific dipeptide interaction previously described here, we have shown in our unpublished work the existence of a thermodynamic molecular switch in the interactions of 35 dipeptides wherein a change of sign in $\Delta Cp^{o}(T)_{reaction}$ leads to true negative minimum in the Gibbs free energy of reaction and hence a maximum in the related $K_{eq}$. Indeed, all interacting biological systems we have examined using the Planck-Benzinger methodology have shown such a thermodynamic molecular switch, suggesting that its existence may be universal[3,4,5,6,7,8,9,10].

## CONCLUSION

The Planck-Benzinger methodology provides a means of determining the innate temperature-invariant enthalpy, $\Delta H^{o}(T_0)$ thermal agitation energy, or the heat capacity integrals, $\int_0^T \Delta Cp^{o}(T)dT$, and allows precise determination of $<T_{Cp}>$, $<T_h>$, $<T_s>$, and $<T_m>$. It is the best method known for evaluating $[\Delta H^{o}_{298} - \Delta H^{o}(T_0)]$, the heat of reaction for biological molecules at room temperature, and provides for a better understanding of cooperative thermodynamic compensation. The Planck-Benzinger methodology demonstrates that macromolecular interactions will always exhibit a negative value of the Gibbs free energy change at a well-defined temperature. It can be used for determination of the thermodynamic molecular switch, where there is a change of sign in $\Delta Cp^o(T)_{reaction}$ which determines the behavior patterns of the Gibbs free energy change.

$$\Delta Cp^o(+) \rightarrow \Delta Cp^o(-) \text{ at low temperature.}$$

All interacting biological systems that we have thus far examined using the Planck-Benzinger approach point to the universality of this thermodynamic switch.

## ACKNOWLEDGMENTS

I wish to thank Dr. Robert J. Hanrahan, Department of Chemistry, University of Florida, for his enlightening discussion and suggestions. I am also grateful to Diane Chun for her valuable suggestions, which have led to an improved manuscript.
